# How Do Career Expectations Affect the Social Withdrawal Behavior of Graduates Not in Employment, Education, or Training (NEETs)? The Chain Mediating Role of Human Capital and Problem-Solving Ability

**DOI:** 10.3390/bs15040506

**Published:** 2025-04-10

**Authors:** Ke Xu, Dandan Zhang, Minghui Wang

**Affiliations:** 1 School of Management, Shanghai University, Shanghai 200444, China; dandanzhang@shu.edu.cn; 2 School of Psychology, Henan University, Kaifeng 475001, China; wmhwang@henu.edu.cn

**Keywords:** graduate not in employment, education, or training (NEET), social withdrawal behavior, career expectations, human capital, problem-solving ability

## Abstract

In recent years, some college graduates in China have chosen to postpone or avoid employment due to the disparity between their career expectations and the reality of the job market, leading to the emergence of a group of graduates not in employment, education, or training (NEETs). As the number of individuals in this group continues to grow, exploring effective strategies to mitigate such behavior has become increasingly important. Based on self-determination theory, this study conducted an empirical analysis using a multi-wave questionnaire survey with a sample of 226 graduate NEETs in Shanghai, China. The findings indicate that (1) career expectations do not directly reduce social withdrawal behavior; (2) the mere accumulation of human capital is insufficient to decrease social withdrawal—only when knowledge and skills are further transformed into problem-solving ability does an individual’s tendency toward social withdrawal significantly decline; and (3) human capital and problem-solving ability serve as a sequential mediating mechanism between career expectations and social withdrawal behavior. This study expands the research on the formation mechanisms of social withdrawal behavior and elucidates the proactive adaptation pathways in individuals’ career adjustment processes. The findings provide theoretical insights for higher education institutions to shift from traditional knowledge transmission models toward a greater emphasis on problem-solving ability development.

## 1. Introduction

The concept of NEET (not in education, employment, or training) was first introduced by the UK government in 1999 to describe adolescents aged 16–18 who, after leaving compulsory education, neither entered the labor market nor pursued further education or vocational training (Furlong, 2006). These individuals, detached early from both education and employment, were found to face a high risk of social exclusion, prompting policy interventions to support their reintegration (Eurofound, 2016). Over time, the European Foundation progressively extended the NEET age range—first to 15–24 in 2012, then to 15–29 in 2016—and developed detailed subcategories to reflect the group’s heterogeneity (Eurofound, 2012, 2016). Among these categories, most NEETs face external or personal barriers (e.g., illness, disability, caregiving duties, or lack of skills) that limit their capacity to re-engage with the labor market (Eurofound, 2016). In contrast, opportunity seekers are willing to work but face difficulties finding jobs that match their qualifications or career expectations (Eurofound, 2012). Due to their distinctive psychological characteristics and greater developmental potential, this group has become a focal point in NEET-related research.

In recent years, this group has grown significantly in China. The National Bureau of Statistics reported that the unemployment rate among Chinese youth aged 16–24 was 14.9% in December 2023, rising to 18.8% by August 2024 (NBSC, n.d.). Among them, the proportion of unemployed college graduates has steadily increased, from 18.9% in 2023 to 19.1% in 2024 (P. Zhao, 2024). These graduates typically possess positive career expectations and social capital; however, they temporarily remain in NEET state due to their inability to secure an ideal job (X. Wang et al., 2022). In academic discourse, they are referred to as graduate NEETs, denoting young individuals with higher education and skills but temporarily unemployed (Afyonoglu et al., 2024).

The causes of graduate NEET status can be understood from three main perspectives. Firstly, from an economic perspective, factors like economic downturns and events such as the COVID-19 pandemic have diminished employment opportunities, which in turn increase the likelihood of graduates becoming NEET (Aina et al., 2024; Kalleberg, 2020). Secondly, from the family perspective, greater family social capital provides financial support, allowing young people to delay employment while seeking ideal jobs. Parents’ high investment in education may also make them less supportive of lower-return career paths (Broschinski et al., 2022; Gauthier & de Jong, 2021; Widyowati et al., 2024). Thirdly, at the individual level, many graduates face skill gaps, low employability, and limited job-search confidence, which—along with increasing social tolerance of NEET status—contribute to their prolonged unemployment (Qian et al., 2024; Yizengaw, 2018; L. Zhao et al., 2024).

Although existing studies have explored various strategies to reduce NEET status, including educational reform, social equity, and individual characteristics (Afyonoglu et al., 2024; Atay & Guneri, 2023; L. Zhao et al., 2024; Zudina, 2022), research remains limited in systematically integrating the cognition–knowledge–ability–behavior framework within a unified model to comprehensively uncover the underlying mechanisms of graduate NEET status from an individual perspective. Therefore, based on self-determination theory, this study aims to systematically explore how career expectations influence the social withdrawal behavior of graduate NEETs. The findings will provide valuable insights for policymakers and guidance for higher education institutions to support graduates in securing meaningful and fulfilling employment.

## 2. Literature Review and Research Hypothesis

### 2.1. Career Expectations and Social Withdrawal Behavior

Social withdrawal behavior, originally a term used to describe children’s tendencies to avoid social interactions (Rubin et al., 2009), has increasingly been extended to adult populations in recent research. Among adults, social withdrawal behavior refers to a negative reaction exhibited by adults when facing setbacks in competitive situations, typically resulting in task abandonment and avoidance (Zheng, 2019). For graduate NEETs, structural constraints in the job market heighten their vulnerability to such setbacks, often leading to delayed employment (X. Wang et al., 2022). Given the strong similarity in withdrawal patterns between graduate NEETs and adults, this study defines social withdrawal behavior in graduate NEETs as a negative response to career-related setbacks, primarily manifested in task abandonment and a tendency toward avoidance.

Career expectations refer to an individual’s realistic assessment of their future career trajectory and potential achievements (Metz et al., 2009). Empirical evidence suggests that individuals’ expectations about their future development play a pivotal role in shaping their career decisions (Jia et al., 2022). These expectations encompass hopes for desirable outcomes, such as income and prestige (Grund & Brock, 2019; Yuan & Woodman, 2010). According to self-determination theory, when individuals perceive career pursuits as valuable and aligned with their psychological needs, extrinsic motivations can be internalized, fostering autonomous motivation and adaptive career behaviors (Deci & Ryan, 2008; Ryan & Deci, 2000). Moreover, individuals with higher career aspirations tend to perform better in professional contexts (Veestraeten et al., 2021). Following this line of reasoning, this study posits that individuals with positive career expectations are more likely to exhibit proactive vocational behaviors and are less prone to retreating into social withdrawal. This leads to our first hypothesis:

**H1.**  *The career expectations of graduate NEETs have a direct negative impact on their social withdrawal behavior.*

### 2.2. The Mediating Role of Human Capital

Human capital includes both macro-level and micro-level dimensions. This study adopts a micro-level perspective, focusing on the sum of knowledge, skills, and physical abilities possessed by workers with economic value (Roy et al., 2018). According to self-determination theory, individuals are self-directed and proactive agents who act to achieve desired outcomes or avoid undesirable ones, with their developmental potential motivating them to engage in activities that enhance their well-being and personal and professional goals (Gagné & Deci, 2005). Given this reasoning, college graduates with positive expectations for the future are likely to enhance their human capital to adapt, compete, and excel in their chosen careers. Furthermore, research has demonstrated that envisioning a positive future can inspire college graduates to improve themselves and pursue vocational training (Oettingen et al., 2005). Since vocational training is a key method for improving human capital (F. Li et al., 2024), positive expectations may lead to proactive efforts to enhance one’s human capital by increasing skills and knowledge. Therefore, this study posits that career expectations are positively associated with human capital.

Higher levels of human capital are associated with stronger motivation to engage in job preparation and proactive employment behavior (Shiyuan et al., 2022), thereby reducing the likelihood of social withdrawal. Within the self-determination theory framework, competence satisfaction is essential for internalization and integration, ultimately facilitating the development of intrinsic motivation (Gagné & Deci, 2005). For individuals, acquiring knowledge and skills through training or learning contributes to enhanced feelings of competence, which in turn fosters autonomous motivation and promotes active vocational behavior while reducing avoidance tendencies (Fernandez & Moldogaziev, 2015). Moreover, individuals with extensive knowledge and practical skills are more likely to secure quality jobs that match their career aspirations rather than withdrawing from the labor market (Yeung & Rauscher, 2014). Therefore, this study posits that human capital is negatively associated with social withdrawal behavior. This leads to our second hypothesis:

**H2.**  *Human capital mediates the relationship between career expectations and the social withdrawal behavior of graduate NEETs.*

### 2.3. The Mediating Role of Problem-Solving Ability

Problem-solving ability is a sophisticated cognitive and operational skill that enables individuals to address challenges when clear solutions are not immediately available. It involves acquiring and applying new knowledge and solving problems creatively by integrating existing knowledge and experience in innovative ways (D’Zurilla et al., 2011). Grounded in self-determination theory, an individual’s positive future expectations foster intrinsic motivation to achieve their goals (Gagné & Deci, 2005). This intrinsic motivation frequently ignites individuals’ interest and encourages them to voluntarily dedicate time to exploring and developing solutions, thereby augmenting their problem-solving ability (Terrazas-Bañales, 2019). Consequently, it can be postulated that individuals with positive career expectations are more likely to proactively improve their problem-solving ability.

Furthermore, research has demonstrated a strong link between problem-solving ability and career preparedness (George & Paul, 2024). According to self-determination theory, the extent to which individuals’ basic psychological needs are satisfied significantly influences their behavioral outcomes (Gagné & Deci, 2005). Individuals with strong problem-solving ability can effectively address challenges, enhancing their sense of competence, which boosts intrinsic motivation and self-efficacy (Erozkan, 2014). These psychological gains foster a greater sense of control, which encourages proactive career decision-making and reduces social withdrawal (Zimmerman et al., 2012). In addition, the satisfaction of the need for competence can further stimulate individuals’ initiative during the career preparation stage, prompting them to engage in positive and optimistic job-seeking behaviors (Mainert et al., 2019). Therefore, this study proposes that problem-solving ability negatively predicts social withdrawal behavior. This leads to our third hypothesis:

**H3.**  *Problem-solving ability mediates the relationship between career expectations and the social withdrawal behavior of graduate NEETs.*

### 2.4. Sequential Mediating Role of Human Capital and Problem-Solving Ability

The above discussion highlights two pathways through which career expectations affect social withdrawal behavior: human capital and problem-solving ability. Building on this foundation, the present study further examines the relationship between human capital and problem-solving ability. To that end, a chain mediation model is constructed to explore how career expectations impact the social withdrawal behavior of graduate NEETs through these mechanisms.

Self-determination theory posits that human beings, as organisms with an inherent tendency toward growth, possess innate psychological needs for self-development and self-determination, particularly the pursuit of autonomy, competence, and relatedness (Gagné & Deci, 2005). For individuals, the accumulation of human capital not only provides the necessary knowledge and skills for problem-solving but also enhances their perceived competence, thereby activating intrinsic motivation. This intrinsic drive, in turn, facilitates the transformation of acquired knowledge into practical capabilities, ultimately improving their problem-solving ability (Groot & Van den Brink, 2000). Furthermore, prior research has shown that knowledge, skills, divergent and convergent thinking, individual motivation, and external environments significantly predict problem-solving ability (Lin & Cho, 2011). Among these factors, domain-specific knowledge and skills have been found to be the strongest predictors of problem-solving ability, suggesting that the more knowledge an individual possesses, the greater their problem-solving ability (Siegfried et al., 2019). Therefore, this study proposes that human capital enhances problem-solving ability, and together, they form a chain mediation pathway through which career expectations influence the social withdrawal behavior of college graduates. This leads to our fourth hypothesis:

**H4.**  *Human capital and problem-solving ability serve as sequential mediators in the relationship between career expectations and the social withdrawal behavior of graduate NEETs.*

**Figure 1:**
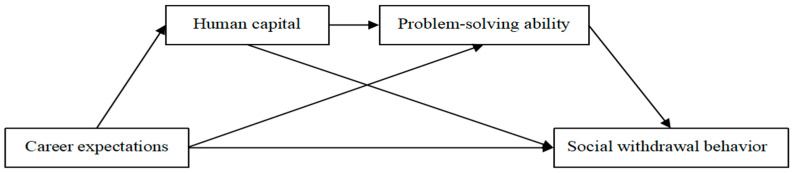
Diagram of the theoretical model.

Based on the above theoretical analysis, this study proposes the research model shown in Figure 1.

## 3. Methods

### 3.1. Participants and Procedure

Participants were selected based on the following criteria: (1) individuals who had completed basic or professional higher education at least six months prior, were currently unemployed, and had been registered as “unemployed college graduates” within the social security system; (2) to exclude individuals engaged in flexible employment, the sample was further limited to those whose main source of livelihood was family support. The questionnaire survey was conducted in three phases from September to November 2023. At Time Point 1, demographic data and data on career expectations were collected; at Time Point 2, data on human capital and problem-solving ability were gathered; and at Time Point 3, data on social withdrawal behavior were recorded.

In the first phase of the study, a total of 260 questionnaires were distributed. After matching and screening across multiple time points, questionnaires with irregular responses were excluded, resulting in a final sample of 226 valid responses. The final response rate was 86.9%. The basic demographic information of the survey sample is presented in Table 1.

### 3.2. Measures

#### 3.2.1. Career Expectations

The career expectations scale was developed with reference to existing measurement tools and was further refined and adapted to fit the specific context of this study (L. Wu & Li, 2001). This scale included five items, which were as follows: “I believe I can gain something beyond money through work”, “I believe that after years of working, I will become a more valuable person to both society and my family”, “I believe that earning my own income allows me to live with greater dignity and independence”, “I believe that work enables me to continuously enhance my abilities rather than merely contributing to a company”, “I believe that my career development depends on my abilities and continuous growth rather than luck”. The Cronbach’s α in this study was 0.794.

#### 3.2.2. Social Withdrawal Behavior

The social withdrawal behavior scale was developed with reference to existing measurement tools and was further refined and adapted to fit the specific context of this study (Coelho et al., 2015). This scale included four items, which were as follows: “I think I can’t cope with complicated things in the workplace”, “I feel nervous and uneasy about the intense competition in the workplace”, “The thought of entering the workplace makes me feel anxious”, “When facing workplace competition and challenges, I often worry that I may not be competent enough”. The Cronbach’s α in this study was 0.717.

#### 3.2.3. Human Capital

The human capital scale was developed with reference to existing measurement tools and was further refined and adapted to fit the specific context of this study (Sharabati et al., 2010). This scale included four items, which were as follows: “I possess the job skills and professional knowledge required by the job position I seek”, “I possess the relevant professional qualifications required for the job position I seek”, “My academic major aligns with the requirements of the job position I seek”, “I am able to communicate with others using industry-specific terms and concepts related to the job position I seek”. The Cronbach’s α in this study was 0.826.

#### 3.2.4. Problem-Solving Ability

The problem-solving ability scale was developed with reference to existing measurement tools and was further refined and adapted to fit the specific context of this study (Frauenknecht & Black, 1995). This scale included three items, which were as follows: “When I encounter difficulties in job hunting, I often try various methods to solve the problems”, “I believe I can effectively handle most challenges encountered in the workplace and find solutions to problems”, “When I disagree with others, I tend to seek solutions through rational communication and collaboration rather than engaging in arguments”. The Cronbach’s α in this study was 0.696.

#### 3.2.5. Control Variables

Gender, age, and education were included as control variables in this study to account for their potential confounding effects on the examined relationships.

## 4. Results

### 4.1. Confirmatory Factor Analysis

This study utilized AMOS version 23.0 (IBM Corporation, Armonk, NY, USA) to perform confirmatory factor analysis and assess the discriminant validity of the core research variables. As shown in Table 2, the four-factor model fit the data better than alternative models (three-factor, two-factor, and single-factor). Thus, the research variables demonstrated good discriminant validity.

### 4.2. Common Method Bias Test

This study used two methods to assess common method bias. Firstly, Harman’s single-factor test was conducted on career expectations, human capital, problem-solving ability, and social withdrawal behavior. Four factors with eigenvalues greater than 1 were extracted, with the first accounting for 29.563% of the variance, below the 40% threshold (Fuller et al., 2016), suggesting that common method bias was not a serious issue. Secondly, the unmeasured latent method construct (ULMC) test showed that adding a common method factor (CMV) did not improve the model fit indices by more than 0.1 (χ^2^ = 121.569, df = 81,χ^2^/df = 1.501, RMSEA = 0.047, CFI = 0.966, GFI = 0.937, IFI = 0.967), further indicating that common method bias was not a significant concern (Howard et al., 2024).

### 4.3. Descriptive Statistics and Correlations

Table 3 presents the means, standard deviations, and correlation coefficients of the variables. Career expectations are significantly positively correlated with human capital (r = 0.291, *p* < 0.01) and problem-solving ability (r = 0.387, *p* < 0.01), while significantly negatively correlated with social withdrawal behavior (r = −0.179, *p* < 0.01). Human capital is positively correlated with problem-solving ability (r = 0.441, *p* < 0.01) and negatively correlated with social withdrawal behavior (r = −0.252, *p* < 0.01). Similarly, problem-solving ability is negatively correlated with social withdrawal behavior (r = −0.343, *p* < 0.01). These results provide preliminary support for this study’s hypotheses.

Regarding demographic variables, gender is positively correlated with human capital (r = 0.183, *p* < 0.01) and problem-solving ability (r = 0.148, *p* < 0.05). Education level is also positively correlated with human capital (r = 0.305, *p* < 0.01) and problem-solving ability (r = 0.189, *p* < 0.01), while negatively correlated with social withdrawal behavior (r = −0.163, *p* < 0.05). These findings highlight the necessity of controlling for demographic variables in subsequent analyses to ensure result robustness.

### 4.4. Hypothesis Testing

This study utilized SPSS version 25.0 (IBM Corporation, Armonk, NY, USA) and the PROCESS macro (version 3.3) developed by Andrew F. Hayes, with gender, age, and education level as control variables, to examine the direct effect of career expectations on social withdrawal behavior among graduate NEETs and the mediating roles of human capital and problem-solving ability. The results are presented in Table 4 and Table 5.

Firstly, the direct effect of career expectations on social withdrawal behavior was tested. The regression results in Table 4 indicated that the direct effect of career expectations on social withdrawal behavior was not significant (β = −0.041, *p* > 0.05). Additionally, the Bootstrap test results in Table 5 showed that the direct effect of career expectations on social withdrawal behavior was −0.041, with a 95% confidence interval of [−0.190, 0.108], which included 0, further confirming the non-significance of the direct effect.

Secondly, the mediating role of human capital was tested. The regression results in Table 4 showed that career expectations positively influenced human capital (β = 0.277, *p* < 0.001). However, human capital did not significantly predict social withdrawal behavior (β = −0.106, *p* > 0.05). Additionally, the Bootstrap analysis results in Table 5 indicated that the indirect effect of career expectations on social withdrawal behavior through human capital was −0.030, with a 95% confidence interval of [−0.086, 0.014], which included 0, confirming the non-significance of the mediating effect.

Thirdly, the mediating role of problem-solving ability was tested. The regression results in Table 4 showed that career expectations positively influenced problem-solving ability (β = 0.273, *p* < 0.001), and problem-solving ability negatively influenced social withdrawal behavior (β = −0.301, *p* < 0.001). Additionally, the Bootstrap analysis results in Table 5 indicated that the indirect effect of career expectations on social withdrawal behavior through problem-solving ability was −0.082, with a 95% confidence interval of [−0.166, −0.024], which excluded 0, confirming the significance of the mediating effect.

Finally, the sequential mediating role of human capital and problem-solving ability was tested. The regression results in Table 4 indicated that career expectations positively influenced human capital (β = 0.277, *p* < 0.001), human capital positively influenced problem-solving ability (β = 0.314, *p* < 0.001), and problem-solving ability negatively influenced social withdrawal behavior (β = −0.301, *p* < 0.001). Additionally, the Bootstrap analysis results in Table 5 indicated that the indirect effect of career expectations on social withdrawal behavior through human capital and problem-solving ability was −0.026, with a 95% confidence interval of [−0.054, −0.008], which excluded 0, confirming the significance of the sequential mediating effect.

**Figure 2:**
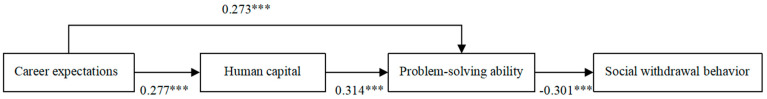
Diagram of the actual model; *** *p* < 0.001.

In summary, Hypotheses 1 and 2 were not supported, whereas Hypotheses 3 and 4 were confirmed. The overall path model of the effect of career expectations on social withdrawal behavior is presented in Figure 2.

## 5. Discussion

### 5.1. Research Conclusions

This study empirically examined the influence of career expectations on the social withdrawal behavior of graduate NEETs through the lens of self-determination theory, and arrived at three key conclusions:

Firstly, career expectations do not exert a significant direct effect on social withdrawal behavior. This suggests that while many graduates may hold high aspirations for their future careers, such expectations alone are insufficient to alter behavioral tendencies—particularly withdrawal in the face of career obstacles. One reason may be that career expectations often remain abstract, focusing on ideal outcomes such as social status, financial rewards, or self-fulfillment (R. Wang et al., 2023), rather than detailing concrete strategies or action plans. Without a clear pathway to achieve these expectations, individuals may lack the sustained motivation or capacity to translate intentions into behavior (Strauss et al., 2012). Furthermore, in the context of China’s increasingly competitive and uncertain job market, even well-formed expectations can become sources of frustration when reality fails to align. When career aspirations are unmet, graduates may experience psychological dissonance, reduced self-efficacy, and emotional fatigue, which may reinforce—rather than alleviate—avoidant behavior.

Secondly, the impact of career expectations on social withdrawal is realized primarily through indirect mechanisms, especially through problem-solving ability. This study finds that individuals with positive career expectations are more likely to develop strong problem-solving ability, which, in turn, significantly reduces their tendency toward social withdrawal. This finding aligns with self-determination theory’s emphasis on competence satisfaction as a driver of autonomous behavior (Gagné & Deci, 2005). When individuals believe they can navigate challenges and find solutions, they gain a sense of control over their career paths, which increases resilience and proactive engagement in job-seeking, thereby buffering against withdrawal.

Thirdly, human capital, although positively associated with both career expectations and problem-solving ability, does not independently reduce social withdrawal behavior. One possible explanation is that our conceptualization focuses on micro-level human capital, emphasizing individual knowledge and methods. However, knowledge can be categorized into explicit and tacit forms (Ozyilmaz, 2020). While higher education enhances explicit knowledge, tacit knowledge—crucial for social adaptation—is typically acquired through workplace experience, observation, and imitation (Ozyilmaz, 2020; M. Wu et al., 2013). As graduate NEETs lack such experience, their limited tacit knowledge may explain why human capital alone does not significantly reduce social withdrawal. This also suggests that merely possessing knowledge or methods is insufficient, as their impact may be limited if they are not effectively applied to real-world problem contexts. Furthermore, the study results reveal a chain mediation effect: career expectations → human capital → problem-solving ability → reduced social withdrawal. This mediation emphasizes that problem-solving ability serves as a crucial bridge in transforming human capital into concrete outcomes, thereby effectively reducing social withdrawal behavior. It underscores that the key is not just the accumulation of skills, but the ability to apply them effectively in complex and uncertain environments.

In summary, this study reveals that the reduction in social withdrawal behavior among graduate NEETs depends less on expectations themselves and more on the individual’s ability to transform those expectations into adaptive capabilities—especially problem-solving ability. This finding provides both theoretical and practical guidance for higher education and policy interventions aimed at strengthening graduates’ employment resilience.

### 5.2. Research Contributions

Theoretically, this study deepens the understanding of how career expectations influence social withdrawal behavior among graduate NEETs by adopting an individual-centered lens. While previous studies have primarily focused on external interventions—such as sociocultural norms, psychological treatment, or social welfare systems (T. M. H. Li & Wong, 2015; Zheng, 2019)—this research highlights the internal mechanisms by which individuals respond proactively to career-related adversity. Specifically, it shows that career expectations can initiate a self-directed adjustment process, driving the development of human capital and problem-solving ability, which in turn helps reduce avoidance behavior. This insight introduces a new theoretical framework—the adaptive individual development perspective—which emphasizes the role of internal competency building and strategic self-regulation in facilitating social and career integration.

Moreover, this study contributes to the theoretical refinement of the classical “cognition–capacity–behavior” model by embedding it within a sequential mediation structure. Rather than treating human capital or problem-solving ability as isolated variables (Thuy Thi Hai et al., 2023; C. Wang et al., 2021), the study demonstrates their dynamic interplay. Human capital fosters problem-solving ability, which subsequently reduces social withdrawal tendencies. This nuanced mediation chain deepens our understanding of how intangible expectations translate into concrete behavioral outcomes.

Practically, the study underscores the urgent need for higher education institutions to move beyond traditional, knowledge-oriented teaching models toward an emphasis on real-world competencies. In particular, fostering students’ problem-solving ability is vital for helping them cope with career uncertainty and adapt to the dynamic labor market. The findings offer empirical support for curricular reforms that prioritize experiential learning, critical thinking, and practical engagement. Additionally, the study provides evidence-based guidance for policymakers to design more targeted interventions—such as structured internships, industry collaborations, and adaptive career education programs—that can support youth in building both the confidence and capabilities needed for meaningful employment.

### 5.3. Practical Implications

Based on the study’s findings, the following strategies are proposed to mitigate social withdrawal behavior among graduate NEETs:

Firstly, help graduates develop realistic career expectations. Students should engage in self-reflection to clarify their interests and goals and form expectations grounded in a clear understanding of the job market. Higher education institutions can support this by offering structured career planning courses and promoting exposure to real-world work settings through internships, university–industry partnerships, and practical training. These experiences help align student expectations with actual career pathways.

Secondly, enhance graduates’ problem-solving ability. Critical thinking and hands-on problem-solving should be cultivated through practical engagement and social experience. Universities should integrate problem-based and project-based learning into curricula and assessment systems to prioritize application over rote knowledge. Strengthening problem-solving competence will better prepare students for workplace complexity and uncertainty.

Thirdly, shift the focus of human capital development from knowledge acquisition to competency building. Educational institutions should emphasize experiential learning—such as case studies, simulations, and fieldwork—to help students internalize industry norms and tacit knowledge. Encouraging participation in internships, research, and skills competitions will further support the transition from academic learning to real-world employability.

### 5.4. Limitations and Future Research Directions

This study has several limitations that should be addressed in future research.

Firstly, the sample was drawn from Shanghai’s social security system, which may limit the generalizability of the findings due to regional differences in economic conditions, urban development, and parental education. Broader geographic sampling is needed to enhance external validity.

Secondly, although this study focused on individual-level mechanisms of social withdrawal, macro-level factors such as economic prosperity and job availability also play crucial roles. Future research should adopt a broader perspective to examine these structural influences.

Thirdly, the study examined only mediating effects, while potential moderators—such as family atmosphere and goal orientation—remain underexplored. For example, a democratic family environment may either foster independence or, conversely, encourage avoidance behaviors. Further research is needed to clarify such dynamics and inform targeted policies aimed at improving youth employment and well-being.

## Figures and Tables

**Table 1:** Basic information of graduate NEETs.

Sample Characteristics	Specific Description	Quantity	Percentage
Gender	Male	99	43.8%
	Female	127	56.2%
Age	18–22	171	75.7%
	22–25	45	19.9%
	25–29	10	4.4%
Education	Vocational college	114	50.4%
	Undergraduate	107	47.3%
	Master	5	2.2%

**Table 2:** Confirmatory factor analysis results.

Model	Factor Content	χ^2^	df	χ^2^/df	RMSEA	CFI	GFI	IFI
Four-factor model	CE; HC; PA; SWB	201.309	97	2.075	0.069	0.912	0.902	0.914
Three-factor model	CE + HC; PA; SWB	481.798	101	4.770	0.129	0.680	0.734	0.685
Two-factor model	CE + HC + PA; SWB	537.891	103	5.222	0.137	0.634	0.715	0.640
Single-factor model	CE + HC + PA + SWB	652.554	104	6.275	0.153	0.539	0.678	0.545

Note: *N* = 226; CE indicates career expectations; HC indicates human capital; PA indicates problem-solving ability; SWB indicates social withdrawal behavior; “+” indicates two factors combined into one factor.

**Table 3:** The mean, standard deviation, and correlation coefficient of research variables.

	M	SD	1	2	3	4	5	6	7
1. Gender	1.560	0.497	1.000						
2. Education	1.520	0.543	0.053	1.000					
3. Age	1.290	0.543	0.090	−0.010	1.000				
4. Career expectations	3.758	0.662	0.067	0.111	−0.119	1.000			
5. Human capital	3.268	0.709	0.183 **	0.305 **	0.044	0.291 **	1.000		
6. Problem-solving ability	3.502	0.664	0.148 *	0.189 **	−0.065	0.387 **	0.441 **	1.000	
7. Social withdrawal behavior	2.792	0.724	−0.017	−0.163 *	0.014	−0.179 **	−0.252 **	−0.343 **	1.000

Note: *N* = 226; * *p* < 0.05, ** *p* < 0.01.

**Table 4:** Analysis of the chained mediation model.

Regression Equation		Fit Coefficient			Significance	
Outcome Variable	Predictive Variable	R	R^2^	F	Β	t
Human capital	Career expectations	0.432	0.187	12.693	0.277	4.201 ***
Problem-solving ability	Career expectations	0.526	0.277	16.841	0.273	4.504 ***
	Human capital				0.314	5.269 ***
Social withdrawal behavior	Career expectations	0.373	0.139	5.912	−0.041	−0.544
	Human capital				−0.106	−1.413
	Problem-solving ability				−0.301	−3.745 ***

Note: *N* = 226; *** *p* < 0.001.

**Table 5:** Bootstrap analysis results.

PATH	Effect	Boot SE	Boot CI Lower Bound	Boot CI Upper Bound
X→Y	−0.041	0.076	−0.190	0.108
X→M1→Y	−0.030	0.025	−0.086	0.014
X→M2→Y	−0.082	0.037	−0.166	−0.024
X→M1→M2→Y	−0.026	0.012	−0.054	−0.008

Note: M1 indicates human capital; M2 indicates problem-solving ability.

## Data Availability

Data supporting the reported results are available from the corresponding author on reasonable request.
